# Supplementary figures and images for: Whole genome linkage disequilibrium maps in cattle
Source: BMC Genet. 2007 Oct 25;8:74. doi: 10.1186/1471-2156-8-74 (PMC2174945; doi:10.1186/1471-2156-8-74)

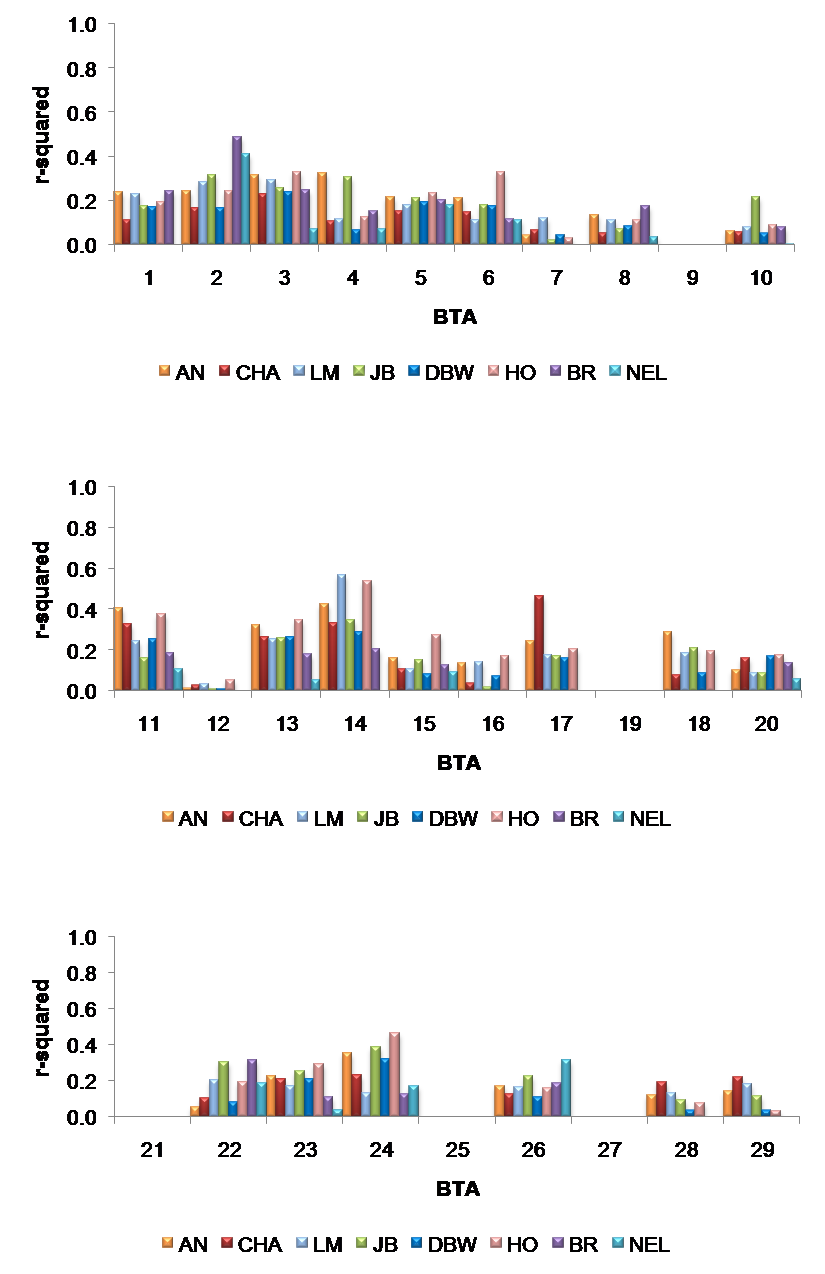

Supplement: Additional file 4 — Average r2 values for inter-marker distances of 5–100 kb for each breed and chromosome. [file 1471-2156-8-74-S4.png]

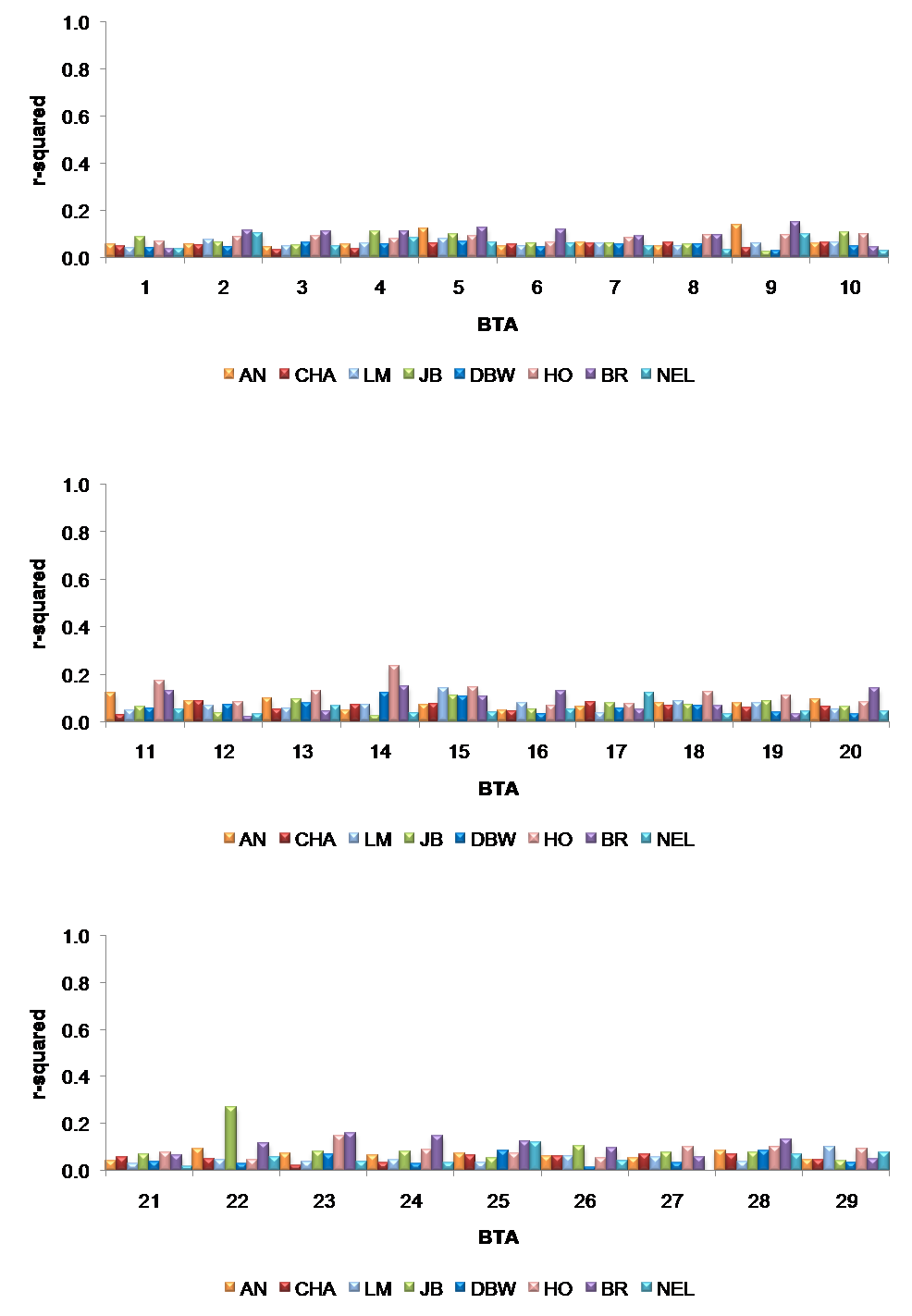

Supplement: Additional file 5 — Average r2 values for inter-marker distances of 100–500 kb for each breed and chromosome. [file 1471-2156-8-74-S5.png]

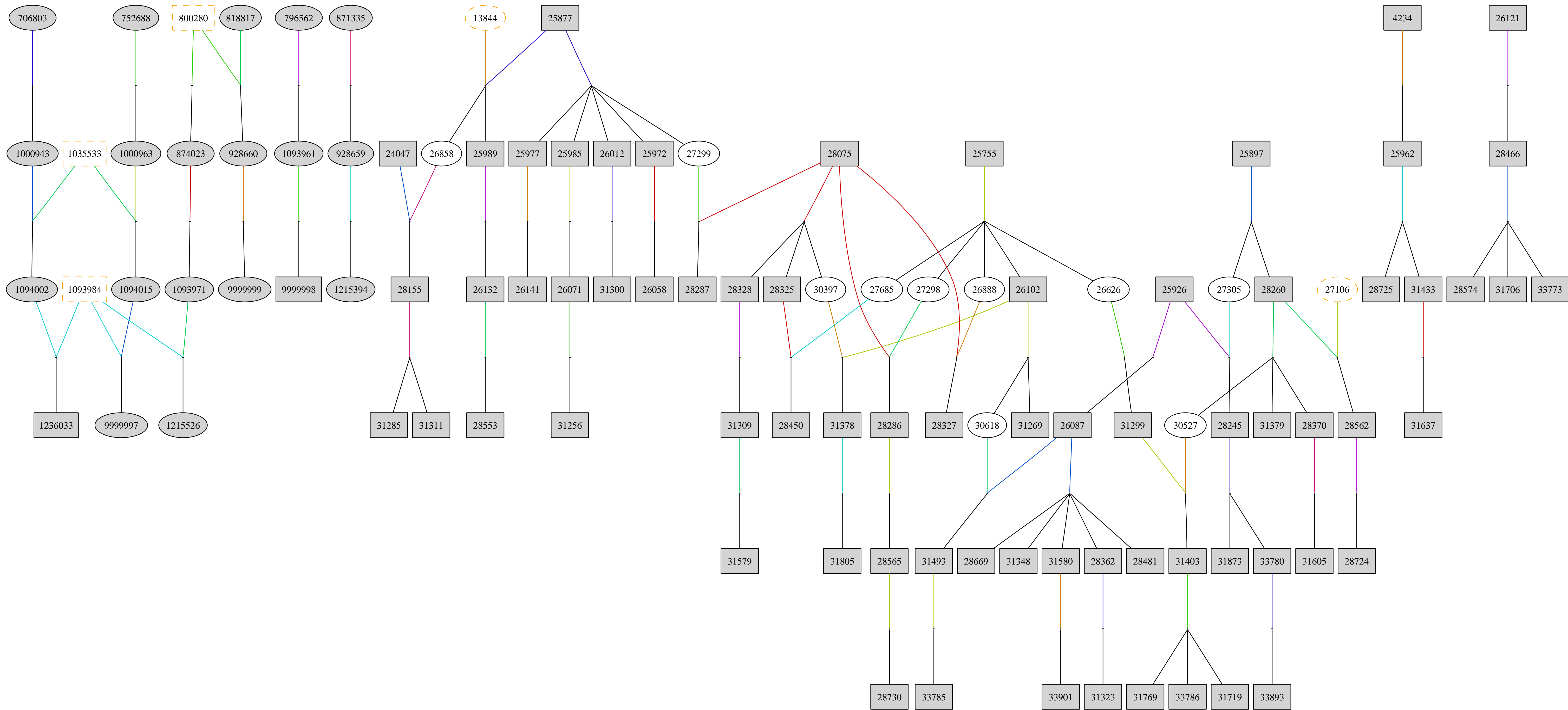

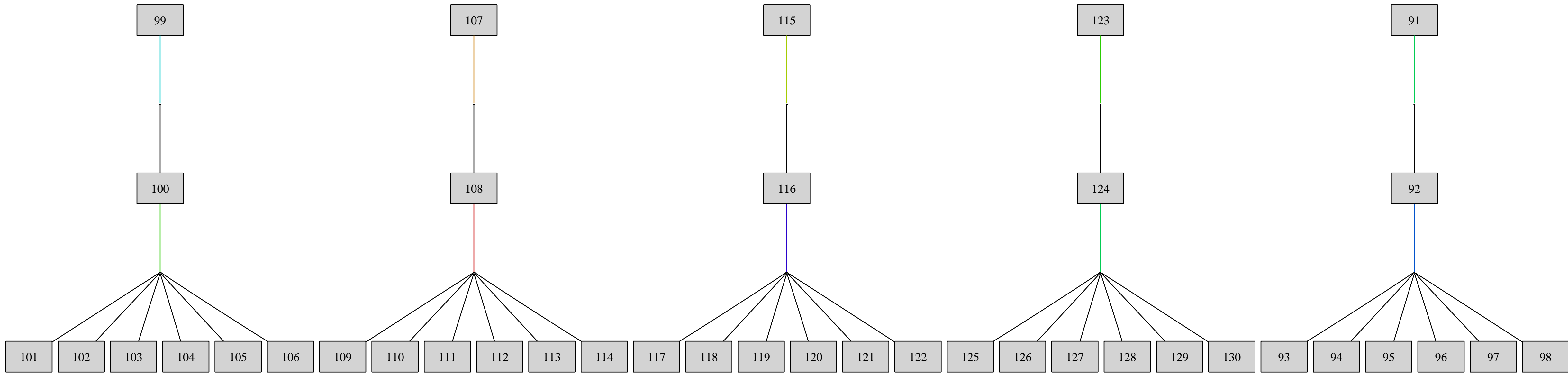

Brahman

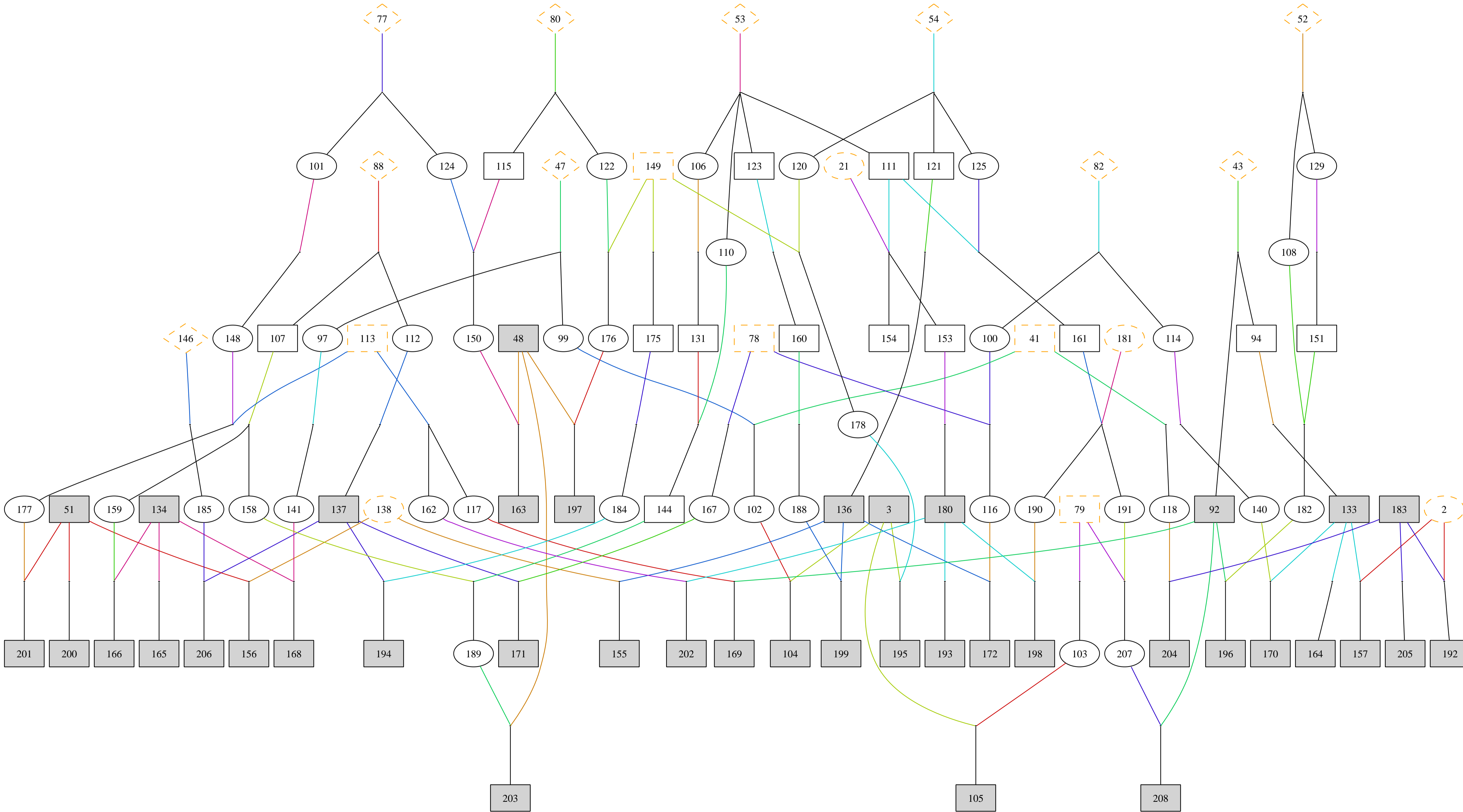

Charolais

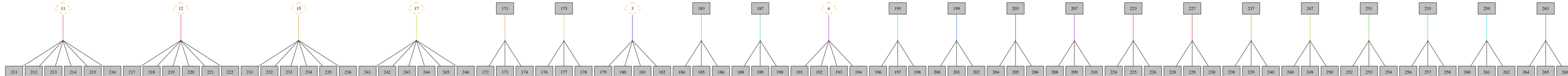

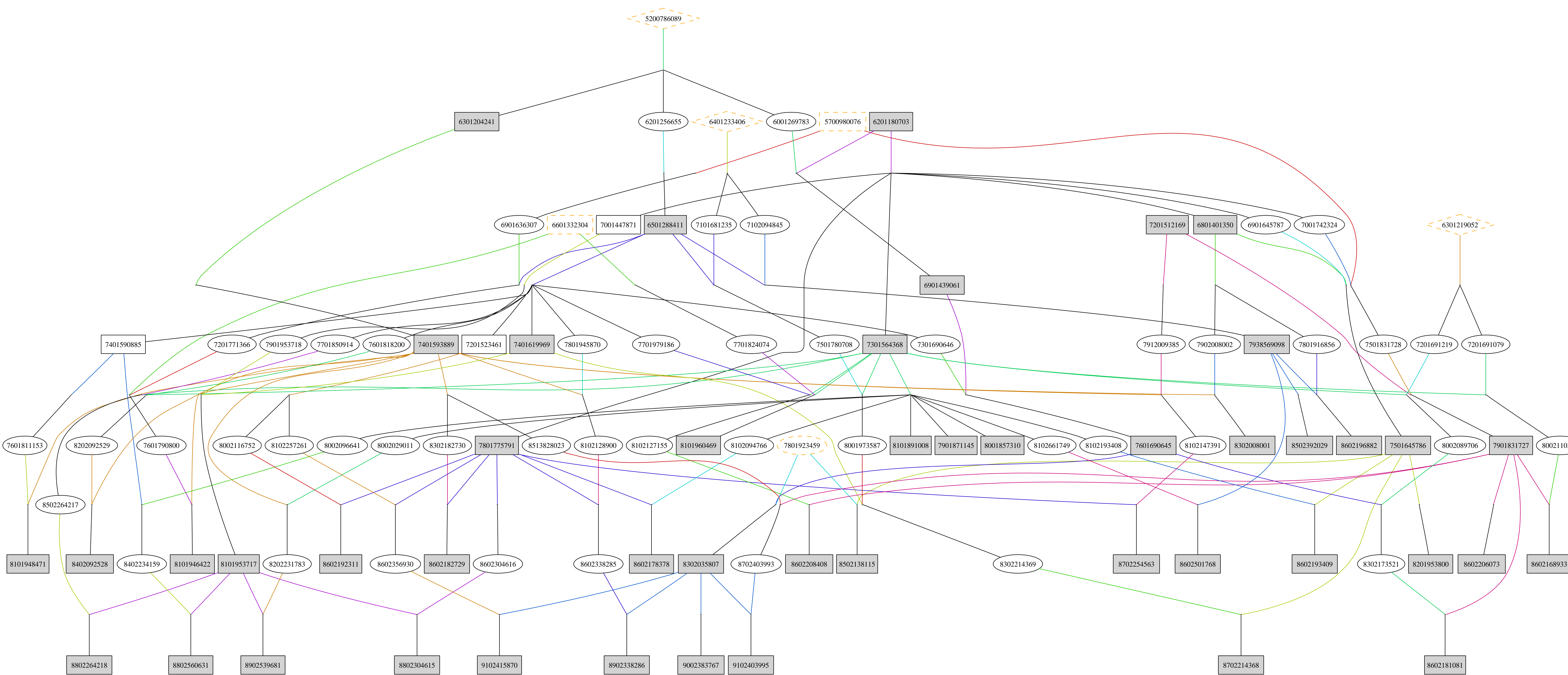

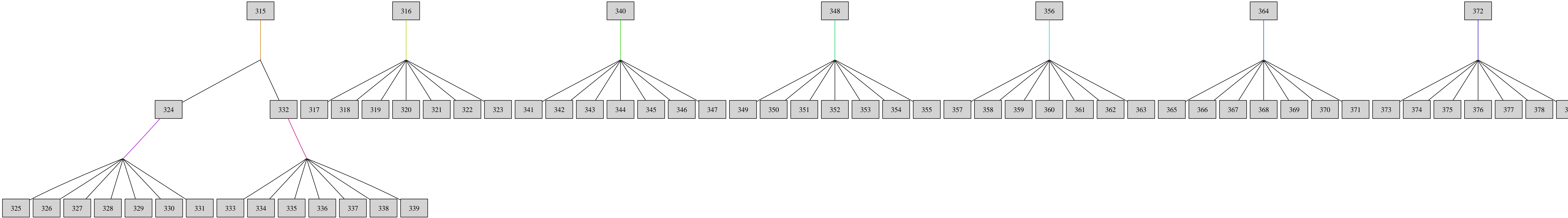

Japanese Black

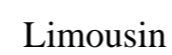

Supplement: Additional file 6 — Pedigrees for each breed. Pedigrees for each breed include a Unique animal IDs, sex of each animal and relationship of each animal genotyped. All genotyped animals were shaded in gray. [file 1471-2156-8-74-S6.pdf]
